# c-Fos enhances influenza virus replication by stabilizing the M2 protein and promoting autophagosome accumulation

**DOI:** 10.1128/jvi.00916-26

**Published:** 2026-07-27

**Authors:** Xiaonan Chen, Junyang Yan, Mengxue Li, Hanbin Liu, Daiqiang Lu, Yang Wang, Yun Liu, Qiao Zhang, Feng Gao, Jiaojiao Peng

**Affiliations:** 1 Institute of Molecular and Medical Virology, School of Medicine, Jinan University 47885, Guangzhou, China; 2 Guangdong Zocode Biotechnology Services Co., Ltd, Guangzhou, China; 3 State Key Laboratory of Respiratory Disease, National Clinical Research Center for Respiratory Disease, National Center for Respiratory Medicine, Guangzhou Institute of Respiratory Health, the First Affiliated Hospital of Guangzhou Medical University 664066, Guangzhou, China; 4 School of Stomatology, Jinan University 47885, Guangzhou, China; 5 Key Laboratory of Viral Pathogenesis & Infection Prevention and Control, School of Medicine, Jinan University 47885, Guangzhou, China; Loyola University Chicago - Health Sciences Campus, Maywood, Illinois, USA

**Keywords:** c-Fos, influenza virus, M2 protein, autophagy, viral replication

## Abstract

The influenza A virus (IAV) employs multiple strategies to hijack host cellular machinery for efficient replication. While autophagosome accumulation is known to promote IAV replication, and the viral matrix protein 2 (M2) ion channel protein plays essential roles in viral uncoating, assembly, and autophagosome accumulation, the mechanisms regulating M2 stability remain incompletely understood. Our previous work established that IAV inhibits SERCA activity to trigger autophagosome accumulation. Here, we identify c-Fos as a critical downstream mediator that stabilizes M2, thereby promoting both autophagosome accumulation and viral replication. Mechanistically, IAV infection drives M2-dependent cytosolic calcium elevation, which upregulates c-Fos expression. The induced c-Fos then binds to M2, protecting it from proteasomal and lysosomal degradation and thereby increasing M2 abundance. This stabilized M2 not only directly enhances viral replication but also sustains autophagosome accumulation to further augment viral growth. Collectively, our findings establish c-Fos as a key regulator of IAV replication via M2 stabilization, revealing a novel mechanism by which the virus amplifies its propagation. These interactions represent potential therapeutic targets for antiviral intervention.

**IMPORTANCE:**

Influenza A virus (IAV) remains a significant global health threat, causing substantial morbidity and mortality. Understanding virus-host interactions is crucial for developing antiviral strategies. Here, we uncover a previously unrecognized self-reinforcing mechanism in which IAV exploits the host transcription factor c-Fos to stabilize its own M2 protein, thereby enhancing viral replication. We demonstrate that IAV infection upregulates c-Fos through M2-mediated calcium signaling, and the induced c-Fos in turn stabilizes the viral M2 protein. This stabilized M2 not only directly supports viral replication but also promotes autophagosome accumulation, further facilitating virus production. Our findings establish c-Fos as a critical proviral host factor and reveal a mechanism by which IAV hijacks host cellular machinery to create a favorable environment for efficient replication. The identification of this c-Fos-M2 axis not only advances our understanding of IAV-host interactions but also opens new avenues for therapeutic intervention targeting this vulnerability in the viral life cycle.

## INTRODUCTION

Influenza A virus (IAV), a member of the Orthomyxovirus family, possesses a lipid envelope and a segmented, negative-sense, single-stranded RNA genome (1). IAV remains a significant global health threat, causing seasonal epidemics and occasional pandemics due to its high mutation rate and zoonotic potential (2, 3). The World Health Organization estimates that annual epidemics result in about 1 billion infections, 3–5 million cases of severe illness, and 290,000–650,000 respiratory deaths globally, underscoring its persistent public health burden (4). Successful viral replication relies on the exploitation of host cellular machinery, with numerous viral and host factors participating in this intricate process. Among these, the IAV M2 protein, an ion channel protein, plays essential roles in viral uncoating, assembly, and budding (5–7). Additionally, M2 has been implicated in inducing autophagy, a conserved cellular process that viruses often hijack to support their replication (8, 9). Despite these critical functions, the mechanisms regulating M2 protein stability remain largely elusive, particularly regarding how host factors modulate its turnover to facilitate viral propagation.

c-Fos, an immediate early gene (IEG)-encoded transcription factor belonging to the activator protein-1 (AP-1) family, plays critical roles in diverse biological processes. It regulates cell proliferation and differentiation and is rapidly induced in response to external stimuli such as oxidative stress, heat shock, and ultraviolet radiation, thereby mediating stress responses (10, 11) and facilitating cellular adaptation to environmental signals (12). Within the immune system, c-Fos participates in the regulation of immune responses by modulating immune cell functions (13). Accumulating evidence suggests that c-Fos is involved in host–virus interactions (14–19). However, whether and how c-Fos modulates IAV replication through the M2 protein remains unknown.

Emerging evidence has established critical roles for Ca²^+^ signaling in IAV replication. Viral entry involves a sialylated voltage-dependent Ca²^+^ channel (20) and a STIM1/Orai1-dependent signaling circuit (21), while intercellular Ca²^+^ wave propagation has been shown to promote infection (22). Beyond entry, IAV infection also induces cytosolic Ca²^+^ imbalances (23). In mammalian cells, sarco/endoplasmic reticulum Ca²^+^-ATPase (SERCA) is an important protein that maintains Ca²^+^ homeostasis by pumping Ca²^+^ from the cytosol into the ER lumen. Inhibition of SERCA activity disrupts this homeostasis, inducing ER stress and influencing both viral infection and autophagy (24–28). We previously demonstrated that SERCA regulates IAV-induced autophagosome accumulation (29). However, the key mediators involved remained unidentified. In this study, we screened for host factors mediating this regulation and identified c-Fos as a critical downstream mediator. We then investigated its role and the underlying mechanism.

Here, we uncover a previously undescribed mechanism by which c-Fos potentiates IAV replication. IAV infection triggers M2-dependent Ca²^+^ influx to upregulate c-Fos, which then binds to M2 and protects it from proteasomal and lysosomal degradation. The resulting M2 stabilization both directly enhances viral replication and sustains autophagosome accumulation to further augment viral growth. Collectively, our findings establish c-Fos as a critical proviral host factor that sustains M2 levels and promotes autophagosome accumulation, revealing a novel regulatory node in the IAV life cycle with therapeutic potential.

## RESULTS

### c-Fos acts as a key downstream mediator of SERCA in IAV infection

**Fig 1:**
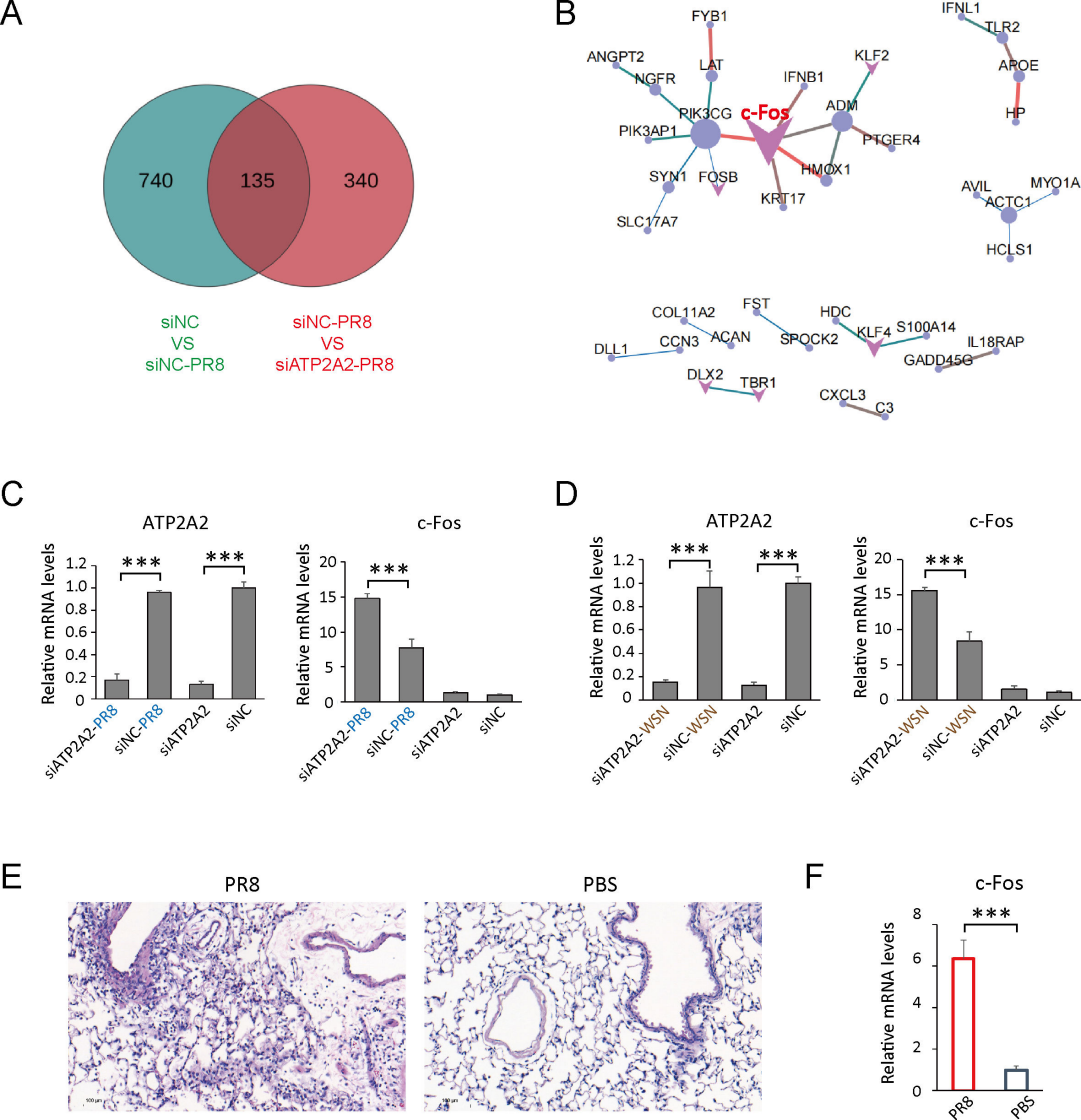
c-Fos acts as a downstream mediator of SERCA in IAV infection. H1395 cells were transfected with siNC or siATP2A2 for 24 h and then infected with PR8/WSN or mock-infected. Total RNA was extracted from the cells and subjected to transcriptomic analysis. (**A**) The Venn diagram shows that green represents the number of genes significantly upregulated by siNC-PR8 relative to the siNC group, red represents the number of genes significantly upregulated by siATP2A2-PR8 relative to siNC-PR8, and the overlapping region in the middle represents the number of genes co-upregulated. (**B**) PPI network showing interactions among common target genes in the Venn diagram. (**C and D**) H1395 cells were transfected with siNC or siATP2A2 for 24 h and then infected with PR8 (**C**) or WSN (**D**), and the transcription levels of ATP2A2 and c-Fos were monitored by qRT-PCR. (**E**) H&E staining of mouse lung tissue. Scale bar, 100 *μ*m. (**F**) mRNA levels of c-Fos in the lung tissue of infected mice were determined by RT-qPCR. All data are presented as means ± SD from at least three independent experiments. Significance was determined by Student’s *t* test. ***, *P*  <  0.001.

We previously showed that IAV infection inhibits SERCA activity and that SERCA knockdown enhances virus-induced autophagosome accumulation (29). To identify the key host factor mediating this effect, we performed transcriptomic analysis on siATP2A2 (SERCA knockdown) and siNC (control) cells that were either mock-infected or infected with the PR8 IAV strain at a multiplicity of infection (MOI) of 1 for 24 h, under conditions in which SERCA knockdown did not compromise cell viability (Fig. S1). Genes upregulated by PR8 infection relative to mock were intersected with those upregulated in siATP2A2-transfected, PR8-infected cells relative to siNC-transfected, PR8-infected controls, yielding 135 candidate host factors (Fig. 1A). Protein-protein interaction (PPI) enrichment analysis identified c-Fos as a central host factor mediating SERCA-dependent effects during IAV infection (Fig. 1B). qPCR validation confirmed that IAV infection upregulated c-Fos transcription, and this effect was further enhanced by SERCA knockdown (Fig. 1C). A similar trend was observed with another influenza strain (WSN) (Fig. 1D).

To further examine the effect of IAV on c-Fos expression *in vivo*, mice were intranasally inoculated with the PR8 strain, and lung tissues were collected three days post-infection. Hematoxylin and eosin (H&E) staining confirmed successful IAV infection and associated inflammatory responses (Fig. 1E). Consistent with the *in vitro* findings, IAV infection significantly upregulated c-Fos expression *in vivo* (Fig. 1F).

Taken together, these results demonstrate that c-Fos acts as a key downstream mediator of SERCA during IAV infection.

### c-Fos promotes IAV-induced autophagosome accumulation and viral replication

**Fig 2:**
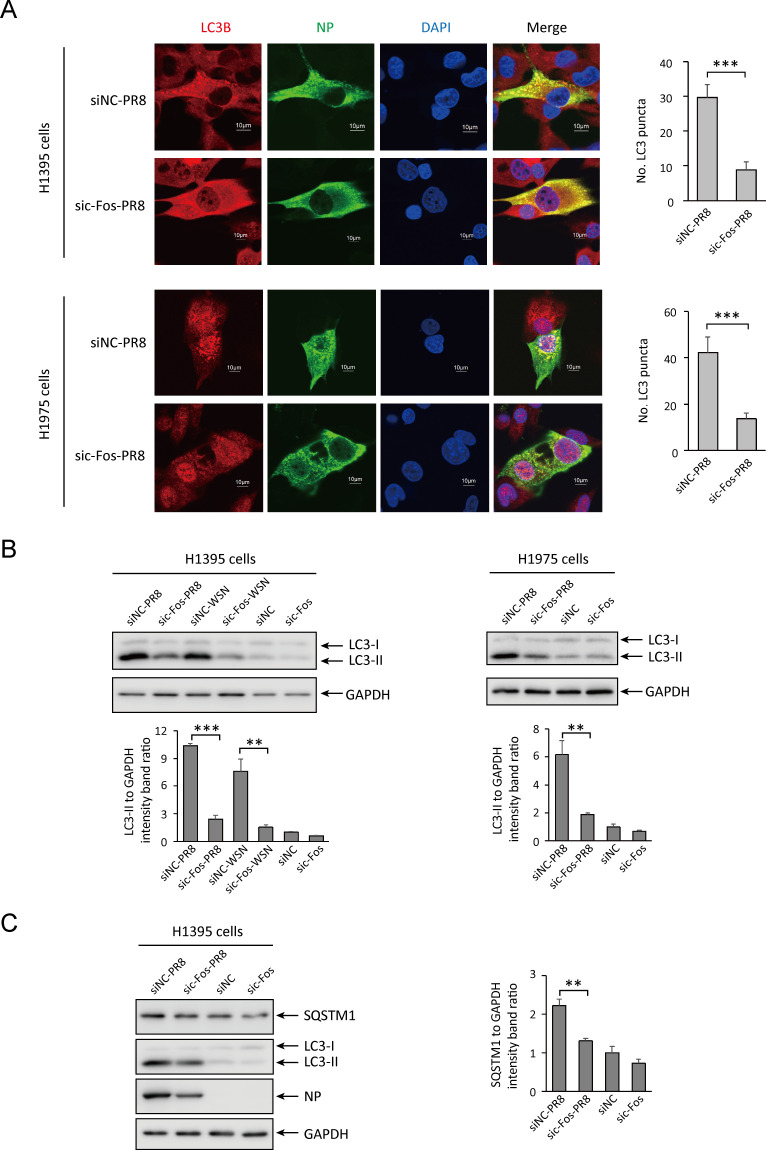
c-Fos enhances IAV-induced autophagosome accumulation. (**A**) Following a 24 h c-Fos knockdown and subsequent PR8 virus infection in H1395 and H1975 cells, the cells were fixed, immunostained with antibodies against LC3 and NP, and examined using confocal microscopy. Scale bar, 10 *μ*m. Quantification of the number of LC3 puncta per cell was measured using a minimum of 10 images per condition. (**B**) H1395 or H1975 cells were transfected with siNC or sic-Fos for 24 h and then infected with PR8, WSN, or mock-infected. The cell lysates were subjected to immunoblotting analysis with the indicated antibodies. (**C**) H1395 cells were transfected with siNC or sic-Fos for 24 h and then infected with PR8 or mock-infected. The cell lysates were subjected to immunoblotting analysis with the indicated antibodies. All data are presented as means ± SD from at least three independent experiments. Significance was determined by Student’s *t* test. **, *P*  <  0.01; ***, *P*  <  0.001.

Since our previous studies demonstrated that SERCA regulates influenza virus-induced autophagy (29), we investigated whether c-Fos, a downstream target of SERCA, modulates this process. In PR8-infected H1395 and H1975 cells, immunofluorescence staining for the viral NP protein and the autophagosome marker LC3B showed that influenza infection robustly induced autophagosome accumulation (Fig. 2A), consistent with prior findings. Notably, c-Fos knockdown in both cell lines significantly reduced this virus-induced autophagosome accumulation (Fig. 2A). To quantify this effect, we measured the conversion of LC3-I to LC3-II, a key step in autophagosome formation. Immunoblot analysis showed that PR8 infection significantly increased the LC3-II/GAPDH ratio compared with mock-infected controls, whereas c-Fos knockdown markedly attenuated this elevation (Fig. 2B), confirming the positive regulatory role of c-Fos in influenza-triggered autophagy. These observations were recapitulated using the WSN viral strain, demonstrating consistent effects across different influenza variants. Collectively, our results establish that c-Fos is involved in promoting influenza virus-induced autophagosome accumulation.

To determine whether c-Fos promotes influenza virus-induced autophagosome accumulation by enhancing its generation or inhibiting their degradation, we assessed the level of SQSTM1. Since SQSTM1 is degraded when autophagic flux is complete, its protein level serves as an indicator of autophagic flux efficiency. Our results showed that infection with influenza virus PR8 upregulated SQSTM1 (Fig. 2C). Knockdown of c-Fos downregulated SQSTM1 (Fig. 2C), indicating restored autophagic degradation, as SQSTM1 is a well-established substrate degraded upon completion of autophagic flux. Consistently, the reduced autophagosome accumulation upon c-Fos knockdown (Fig. 2A and B) is thus attributable to enhanced autophagic flux rather than decreased autophagosome formation. Collectively, these findings suggest that c-Fos knockdown alleviates influenza virus-induced autophagic impairment by promoting autophagic flux.

**Fig 3:**
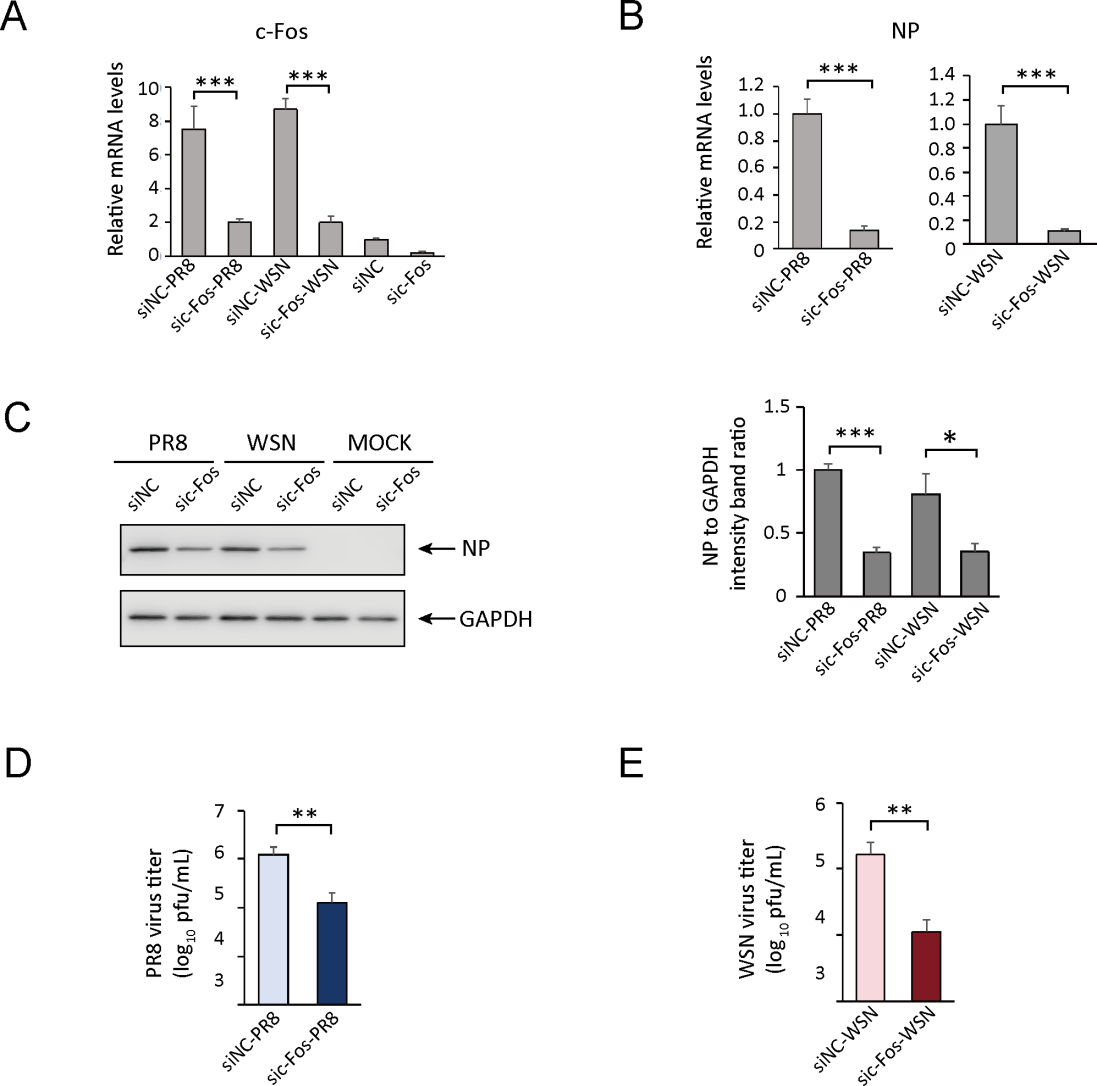
c-Fos promotes IAV replication in H1395 cells. H1395 cells were transfected with siNC or sic-Fos for 24 h and then either mock-infected or infected with PR8 or WSN virus. (**A and B**) qRT-PCR analysis of c-Fos (**A**) and viral NP (**B**) mRNA levels. (**C**) Immunoblot analysis of cell lysates using the indicated antibodies. (**D and E**) Viral titers in supernatants collected 24 h post-infection with PR8 (**D**) or WSN (**E**), determined by plaque assay. All data are presented as means ± SD from at least three independent experiments. Significance was determined by Student’s *t* test. *, *P*  <  0.05; **, *P*  <  0.01; ***, *P*  <  0.001.

Given that autophagic accumulation facilitates IAV replication (30), we reasoned that c-Fos promotes viral replication by enhancing IAV-induced autophagosome accumulation. Consistent with our hypothesis, in H1395 lung cells, infection with PR8 or WSN upregulated c-Fos, whereas c-Fos knockdown suppressed viral gene expression (Fig. 3A through C) and reduced viral titers by 10- to 14-fold (Fig. 3D and E). Similar results were obtained in H1975 cells (Fig. S2A through D). To extend these findings to seasonal influenza, we performed similar knockdown experiments using the H3N2 strain A/Hong Kong/8/68, which also led to a significant reduction in viral replication (Fig. S3A through C). Collectively, these results establish c-Fos as a critical host factor for efficient IAV replication. This conclusion is further supported by recent independent studies demonstrating that c-Fos promotes IAV replication in A549 cells (18).

### IAV promotes c-Fos expression by inducing cytosolic calcium elevation via its M2 protein

**Fig 4:**
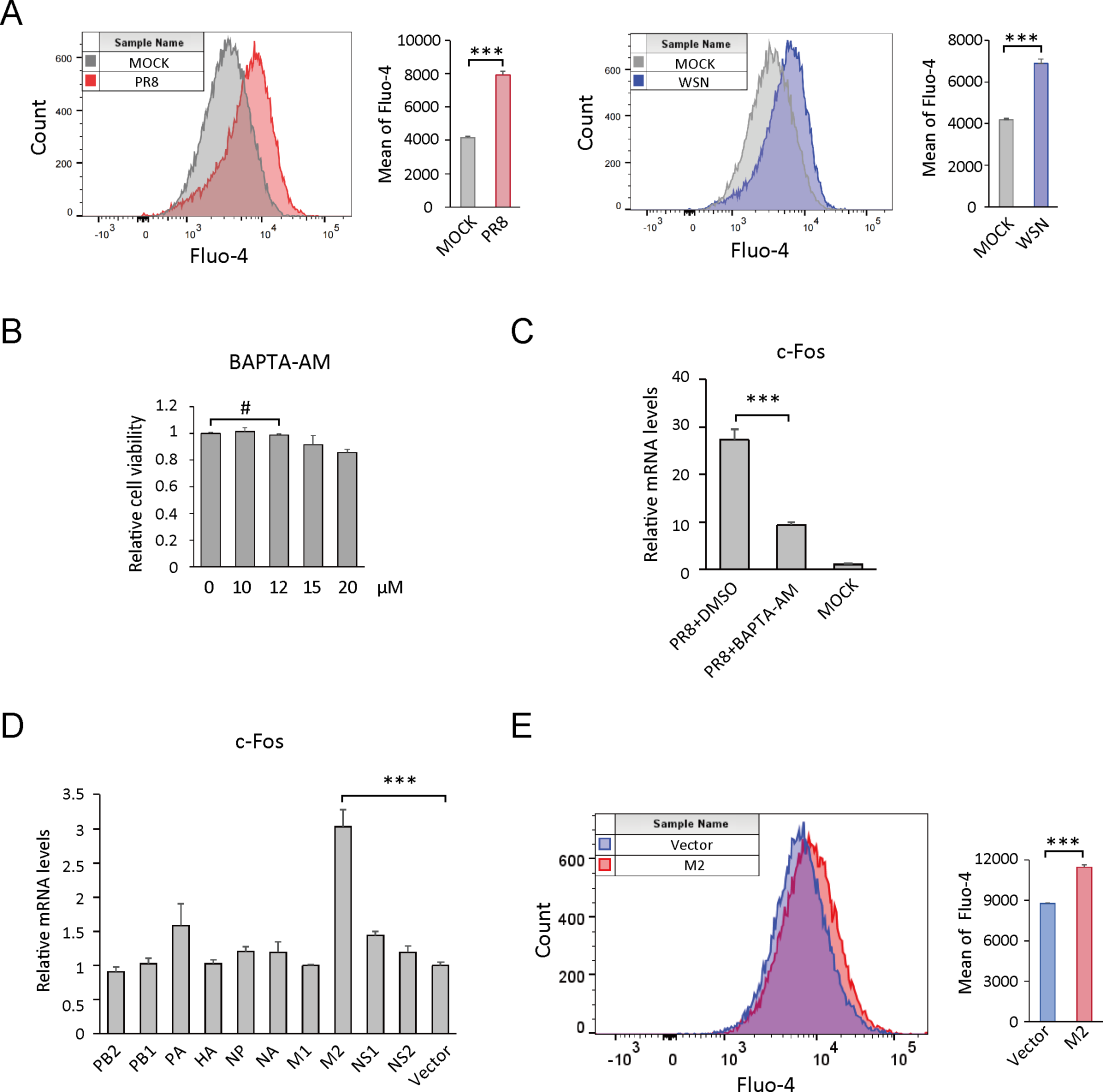
IAV promotes c-Fos expression by inducing cytosolic calcium elevation via its M2 protein. (**A**) H1395 cells were infected with PR8, WSN, or mock-infected. Cytosolic Ca²^+^ was labeled with Fluo-4, and fluorescence intensity was measured by flow cytometry, with 30,000 events collected per sample for analysis. (**B**) Cell viability was assessed by CCK-8 assay in BAPTA-AM-treated cells. (**C**) H1395 cells were pretreated with BAPTA-AM or mock for 2 h, followed by PR8 virus infection. At 24 h post-infection, c-Fos transcription levels were measured by qRT-PCR. (**D**) 293H cells were transfected with plasmids encoding individual viral proteins of the IAV PR8 strain (empty vector as the control). Cells were harvested 48 h post-transfection, and c-Fos transcription levels were analyzed by qRT-PCR. (**E**) 293H cells were transfected with the PR8-M2 plasmid or the empty vector. After 48 h, cytosolic Ca²^+^ was labeled with Fluo-4, and fluorescence intensity was measured by flow cytometry, with 30,000 events analyzed per sample. All data are presented as means ± SD from at least three independent experiments. Significance was determined by Student’s *t* test: ***, *P* < 0.001; #, *P* > 0.05.

Our previous studies showed that influenza virus infection inhibits SERCA activity (29), a calcium pump critical for pumping calcium ions from the cytosol into the endoplasmic reticulum (28), and that SERCA knockdown enhances IAV-induced c-Fos upregulation (Fig. 1C and D). Based on these findings, we hypothesized that IAV elevates c-Fos expression by increasing cytosolic calcium levels. To test this, we first measured cytosolic calcium changes upon infection with PR8 or WSN strains using the fluorescent indicator Fluo-4. Flow cytometry analysis revealed increased Fluo-4 fluorescence in both PR8- and WSN-infected cells (Fig. 4A), indicating that IAV infection elevates cytosolic calcium. To determine whether this calcium flux is responsible for c-Fos induction, we treated cells with BAPTA-AM (12 *µ*M, a non-cytotoxic concentration; Fig. 4B) to chelate cytosolic calcium. Blocking calcium elevation markedly reduced IAV-triggered c-Fos expression (Fig. 4C), supporting our hypothesis that IAV upregulates c-Fos through calcium signaling.

We next sought to identify the viral factor mediating this effect. By individually expressing each of the 10 IAV proteins and assessing their ability to induce c-Fos, we found that the M2 protein alone was sufficient to drive c-Fos transcription (Fig. 4D). M2 expression also elevated cytosolic calcium levels (Fig. 4E), suggesting that M2 promotes c-Fos expression specifically through calcium signaling.

**Fig 5:**
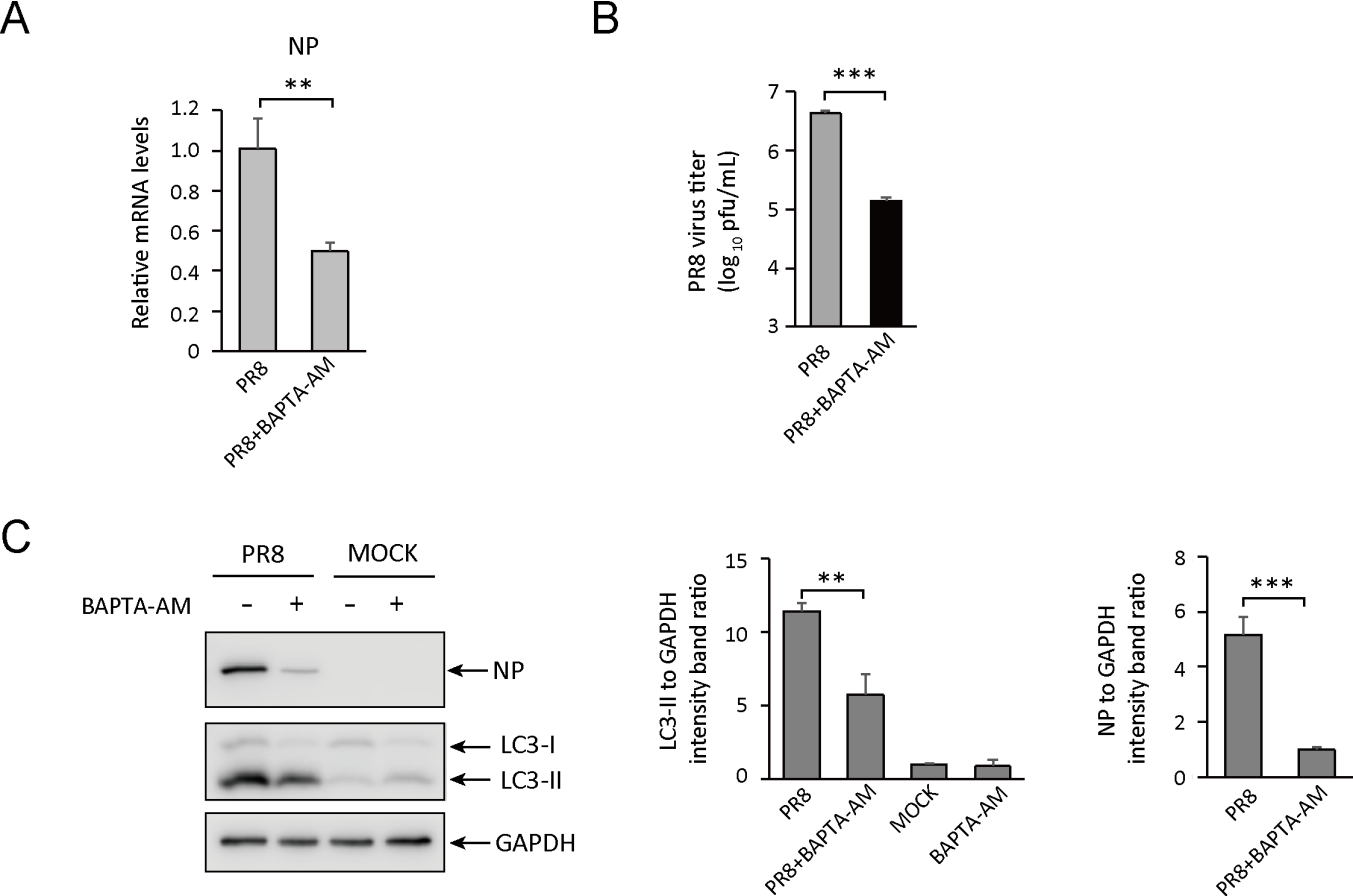
Chelation of cytosolic calcium suppresses IAV replication and virus-triggered autophagy. (**A**) H1395 cells were pretreated with BAPTA-AM or DMSO for 2 h, followed by PR8 virus infection. NP mRNA levels were quantified by qRT-PCR at 24 h post-infection. (**B**) Viral titers in the culture supernatants were determined by plaque assay at 24 h post-infection. (**C**) H1395 cells were pretreated with BAPTA-AM or mock for 2 h, followed by PR8 virus or mock infection. Cell lysates were analyzed by immunoblotting with the indicated antibodies. All data are presented as means ± SD from at least three independent experiments. Significance was determined by Student’s *t* test: **, *P*  <  0.01; ***, *P*  <  0.001.

Having established that c-Fos suppression inhibits IAV-induced autophagosome accumulation (Fig. 2) and IAV replication (Fig. 3), we next examined the role of calcium signaling in these processes. Treatment with the calcium chelator BAPTA-AM reduced c-Fos levels, attenuated viral replication (Fig. 5A through C), and reduced autophagosome accumulation (Fig. 5C). These findings suggest that calcium signaling, likely upstream of c-Fos, contributes to IAV replication and autophagy.

Collectively, these results demonstrate that IAV promotes c-Fos expression by inducing cytosolic calcium elevation through its M2 protein.

### c-Fos stabilizes IAV M2 protein by protecting it from proteasomal and lysosomal degradation

**Fig 6:**
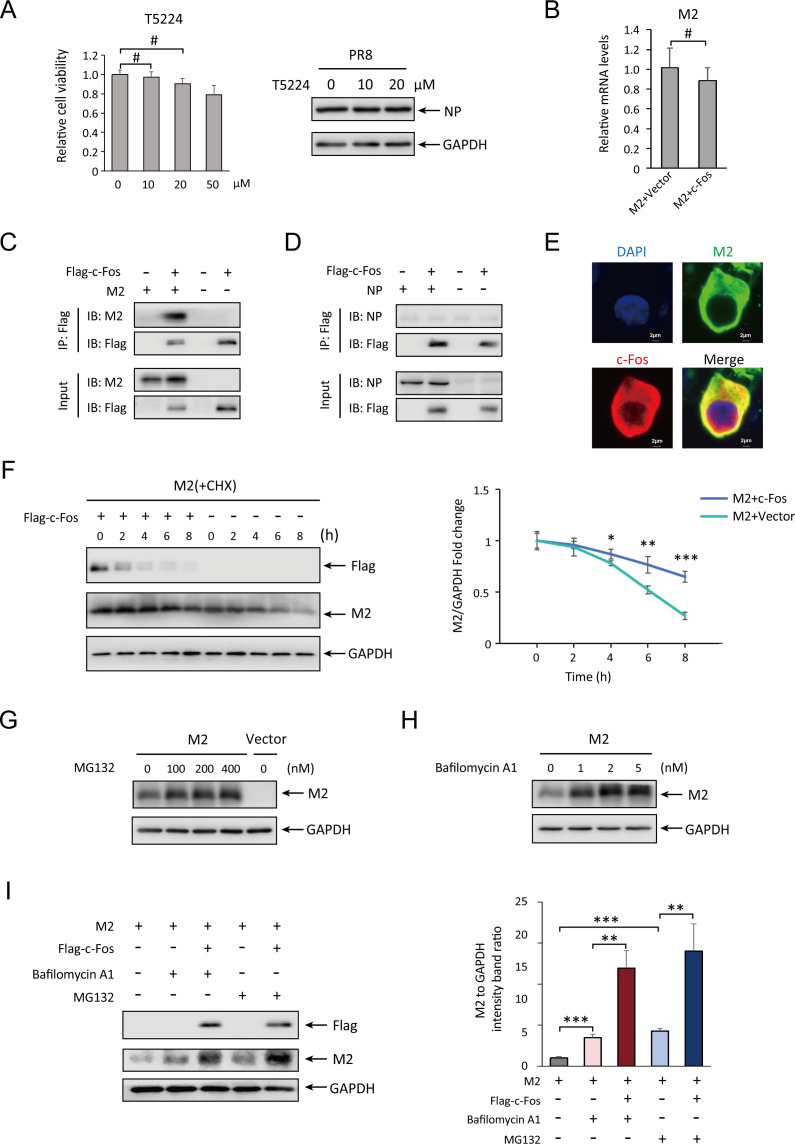
c-Fos stabilizes IAV M2 protein by protecting it from proteasomal and lysosomal degradation. (**A**) Cell viability was assessed by CCK-8 assay following T5224 treatment. For infection experiments, cells were pretreated with 0, 10, or 20 *μ*M T5224 for 2 h before PR8 virus infection. Protein lysates were harvested at 24 h post-infection and analyzed by Western blot using anti-NP and anti-GAPDH antibodies. (**B**) 293H cells were co-transfected with M2 and c-Fos plasmids, alongside M2 and vector controls. After 48 h, cells were lysed and RNA was extracted for RT-qPCR analysis of M2 mRNA expression. (**C and D**) Co-immunoprecipitation and immunoblot analysis of 293H cells co-transfected with Flag-c-Fos and M2 (**C**) or NP (**D**). (**E**) 293H cells were co-transfected with Flag-c-Fos and M2 for 48 h, followed by immunostaining for Flag (red) and M2 (green). Representative confocal immunofluorescence images are shown. Scale bars, 2 *μ*m. (**F**) 293H cells were co-transfected with M2 and Flag-c-Fos or empty vector. Cells were treated with 50 *μ*g/mL CHX and harvested at the indicated time points (0–8 h). M2 protein levels were analyzed by Western blot. (**G and H**) 293H cells were transfected with M2 or empty vector. At 6 h post-transfection, cells were treated with MG132 (100, 200, 400 nM), Bafilomycin A1 (1, 2, 5 nM), or a DMSO control. After 48 h, M2 protein expression was assessed by Western blot. (**I**) 293H cells were co-transfected with M2 and Flag-c-Fos or vector, followed by treatment with MG132 (200 nM) or Bafilomycin A1 (2 nM) for 48 h. M2 protein levels were analyzed by Western blot. All data are presented as means ± SD from at least three independent experiments. Significance was determined by Student’s *t* test: *, *P*  <  0.05; **, *P*  <  0.01; ***, *P*  <  0.001. #, *P* > 0.05.

As c-Fos is a known transcription factor, we first investigated whether its enhancement of IAV replication is dependent on its transcriptional activity. Using T5224, a specific inhibitor of this activity, we pretreated cells with non-cytotoxic concentrations (10 *µ*M and 20 *µ*M) for 2 h and then infected them with the PR8 virus at an MOI of 1 for 24 h in the presence of DMSO or T5224 and observed no significant effect on IAV NP protein expression (Fig. 6A). This result indicates that c-Fos promotes IAV replication through a transcription-independent mechanism. Since we previously identified that IAV upregulates c-Fos expression via its M2 protein, we next examined whether c-Fos reciprocally regulates the M2 protein. Co-transfection experiments with Flag-tagged c-Fos and PR8-M2 plasmids revealed that c-Fos increased M2 protein levels, as shown in the input lanes of Fig. 6C, without affecting M2 mRNA expression (Fig. 6B). This observation led us to hypothesize that c-Fos might interact with M2 to prevent its degradation. Co-immunoprecipitation (Co-IP) assays confirmed a specific interaction between c-Fos and M2 protein (Fig. 6C), while other viral proteins such as NP showed neither binding to c-Fos nor any effect on its abundance (Fig. 6D). Supporting these findings, confocal microscopy revealed clear co-localization of c-Fos with M2 in cells (Fig. 6E). Additionally, cycloheximide (CHX) chase analysis revealed that c-Fos overexpression prolonged the half-life of M2 (Fig. 6F). These results collectively indicate that c-Fos stabilizes M2 protein by protecting it from degradation.

To determine how c-Fos regulates the degradation of M2 protein, we first investigated the pathways responsible for M2 degradation. Using the proteasome inhibitor MG132 and the lysosome inhibitor Bafilomycin A1, we observed that M2 is degraded through both the proteasomal and lysosomal pathways (Fig. 6G and H). Next, to elucidate whether c-Fos inhibits M2 degradation via these pathways, we co-transfected c-Fos and M2 while separately blocking each degradation mechanism. When proteasomal degradation was inhibited by MG132, c-Fos overexpression further reduced M2 degradation, suggesting that c-Fos protects M2 from lysosomal degradation (Fig. 6I). Similarly, our results revealed that c-Fos also reduced proteasomal degradation of M2 (Fig. 6I).

Taken together, these results demonstrate that c-Fos enhances M2 protein stability by simultaneously protecting M2 from both lysosomal and proteasomal degradation.

### c-Fos-M2 axis enhances autophagy to promote viral replication

**Fig 7:**
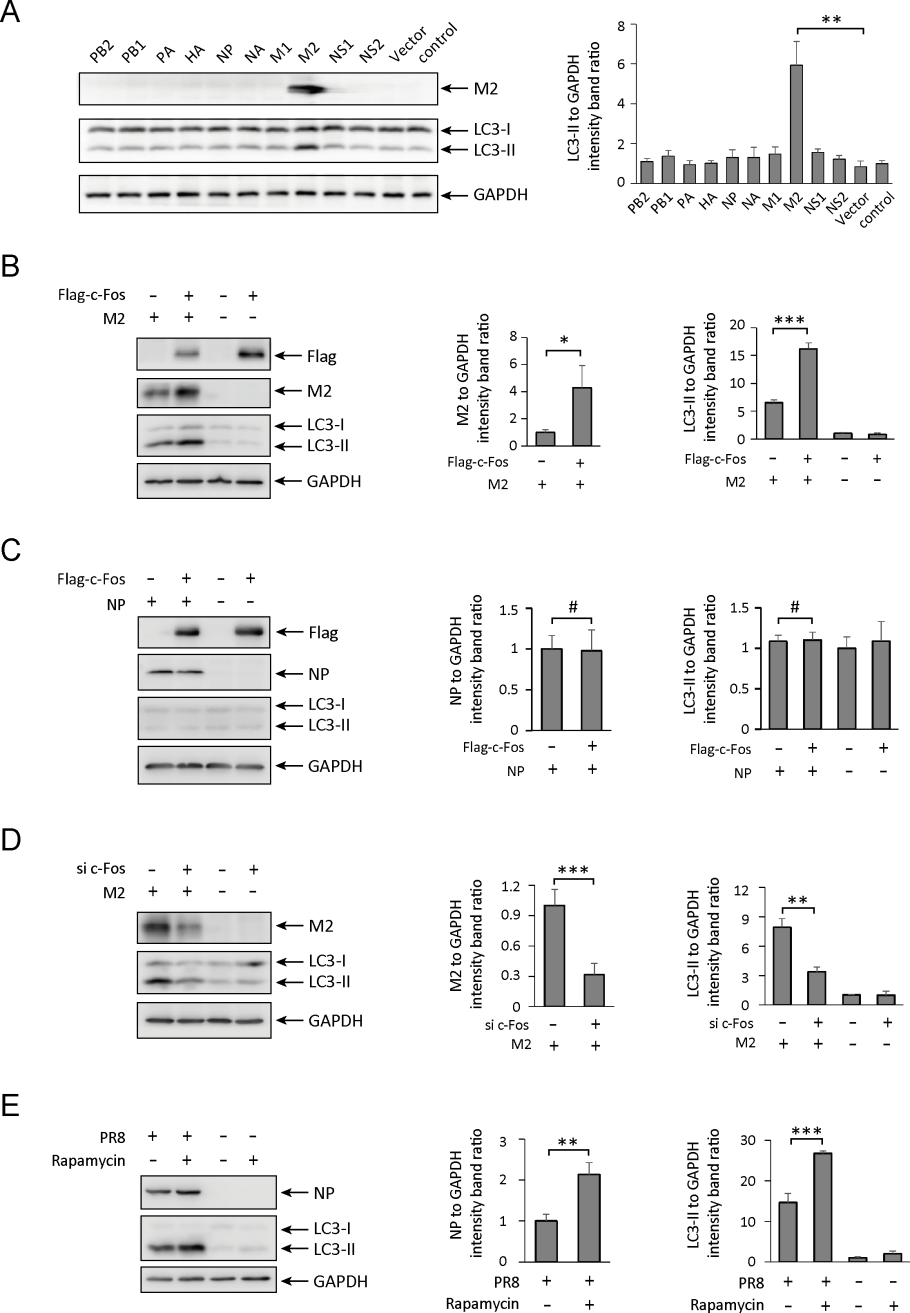
c-Fos promotes IAV replication by stabilizing M2 protein to induce proviral autophagy. (**A**) 293H cells were transfected with plasmids encoding individual viral proteins of the IAV PR8 strain. An empty vector was used as a negative control. Cells were harvested 48 h post-transfection, and lysates were analyzed by Western blotting to assess LC3 lipidation (LC3-II) levels, with GAPDH used as a loading control. (**B and C**). 293H cells were co-transfected with Flag-c-Fos and M2 (**B**) or NP (**C**) plasmids. An empty vector was used as a control. Cells were harvested 48 h post-transfection and analyzed by Western blotting for the indicated proteins. (**D**) 293H cells were transfected with siRNA targeting c-Fos (sic-Fos) or negative control siRNA (siNC). At 24 h post-siRNA transfection, cells were further transfected with the M2 plasmid and harvested 48 h later. Protein levels were analyzed by Western blotting. (**E**) H1395 cells were pretreated with 100 nM rapamycin or an equivalent volume of DMSO for 6 h, followed by infection with PR8 virus (MOI = 1) or mock infection. Cells were harvested 12 h post-infection and analyzed by Western blotting for viral NP and LC3-II levels. All data are presented as means ± SD from at least three independent experiments. Significance was determined by Student’s *t* test: *, *P*  <  0.05; **, *P*  <  0.01; ***, *P*  <  0.001. #, *P* > 0.05.

Building upon our previous demonstration that c-Fos contributes to the promotion of both virus-induced autophagosome accumulation and IAV replication, we sought to elucidate the underlying mechanism. Since M2 is not only essential for IAV replication but has also been shown to be the key viral protein driving autophagosome accumulation, and given the established role of autophagy in promoting influenza infection, we hypothesized that c-Fos enhances IAV replication by stabilizing M2 protein, thereby promoting autophagosome accumulation. To test this hypothesis, we first confirmed M2’s specific role in IAV-induced autophagy in H1395 cells. Expression of all 10 IAV proteins individually revealed that only PR8 M2 protein could independently increase levels of the autophagosome marker LC3-II (Fig. 7A). Importantly, c-Fos overexpression enhanced both M2 protein levels and LC3-II accumulation, while showing no such effect on IAV NP protein (Fig. 7B and C). Conversely, RNAi-mediated knockdown of c-Fos reduced both M2 protein stability and LC3-II levels (Fig. 7D). These results demonstrate that c-Fos enhances virus-induced autophagosome formation, specifically through M2 stabilization.

To establish the functional link between M2-induced autophagy and viral replication, we treated cells with the autophagy inducer rapamycin prior to IAV infection. Enhanced autophagosome accumulation significantly increased viral replication (Fig. 7E). Taken together, our findings reveal that c-Fos stabilizes the M2 protein by protecting it from degradation, which subsequently enhances virus-induced autophagosome formation, thereby promoting influenza virus replication.

## DISCUSSION

**Fig 8:**
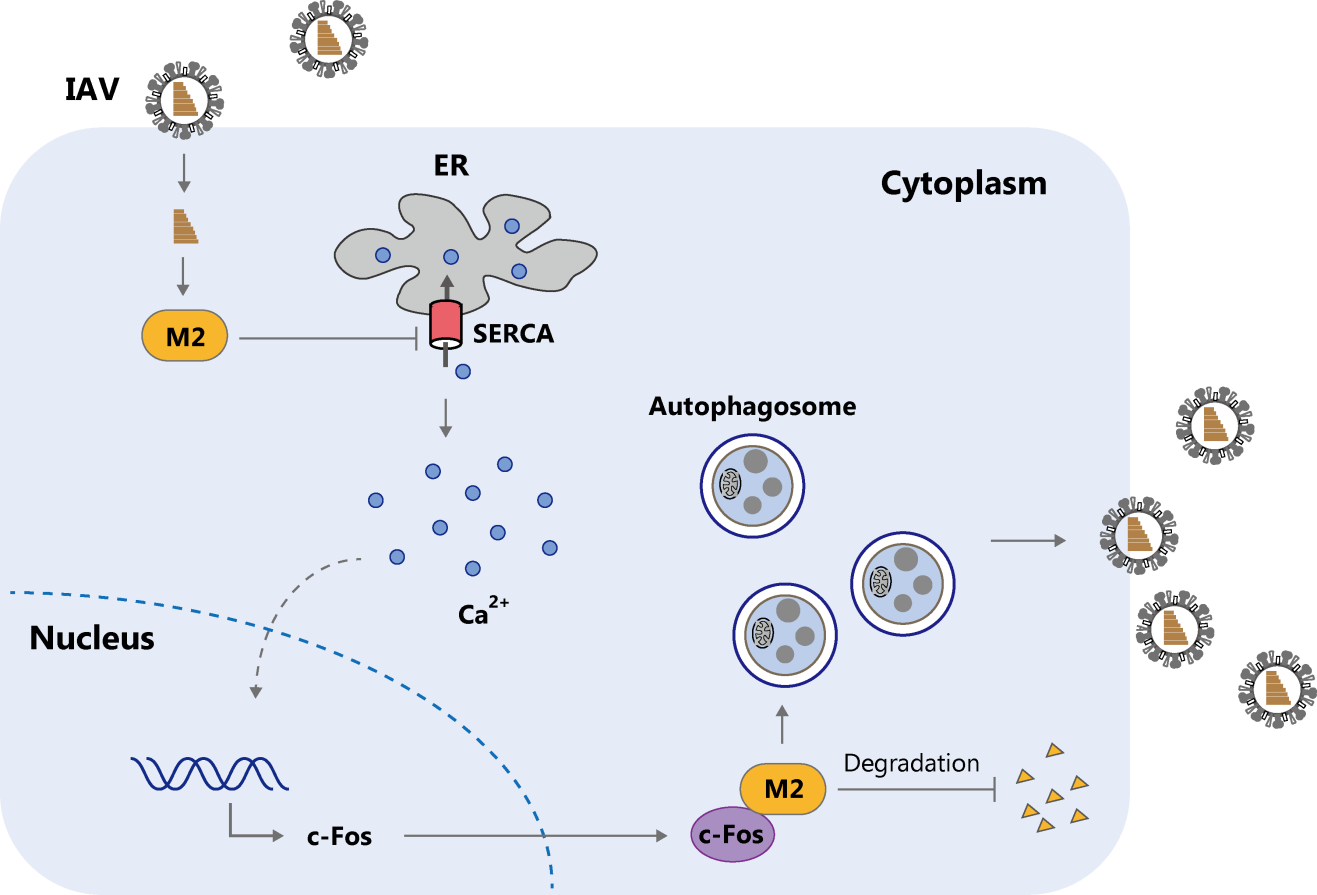
Schematic diagram of a model for the mechanism by which c-Fos promotes IAV replication. Following influenza virus infection, the viral M2 protein inhibits the host SERCA pump, resulting in disrupted calcium homeostasis and elevated cytosolic calcium. This calcium elevation induces c-Fos upregulation, which consequently interacts with M2 and inhibits its degradation. The stabilized M2 thereby promotes virion assembly and budding, while also facilitating autophagosome accumulation to create a favorable intracellular environment for viral replication.

IAV poses a significant threat to human health by triggering severe respiratory inflammation that can progress to acute lung injury and mortality. Understanding virus-host protein interactions is therefore critical for uncovering novel antiviral strategies. Our study elucidates a key mechanism centered on the host transcription factor c-Fos. We identify c-Fos as a proviral regulator that enhances IAV replication. Specifically, IAV infection induces M2-mediated calcium influx leading to c-Fos upregulation, and c-Fos in turn stabilizes M2 protein and promotes autophagosome accumulation, thereby amplifying viral propagation (Fig. 8).

Our study elucidates the complex interplay between IAV infection and autophagy. While it is well-established that IAV infection both induces autophagy initiation and inhibits autophagosome maturation (30), with the M2 protein being particularly implicated in autophagosome accumulation (8, 31–33), we identified the c-Fos-M2 axis as a key regulator of autophagosome accumulation that enhances viral replication. We demonstrated that M2 alone can induce LC3-II accumulation (Fig. 7A), an effect that is significantly amplified by c-Fos-mediated M2 stabilization (Fig. 7B through D). Notably, we found that c-Fos regulates autophagosome accumulation through additional mechanisms beyond M2 stabilization. Knockdown of c-Fos enhanced autophagic flux and promoted autophagosome-lysosome fusion even in uninfected cells (Fig. 2C), revealing its multifaceted role in autophagy regulation. While previous studies have shown c-Fos can induce autophagy through upregulation of BECN1 (34, 35), supporting our findings, the precise mechanisms governing c-Fos-mediated autophagy regulation in IAV-susceptible pulmonary cells require further investigation due to differences in experimental models.

Ca^2+^ is a ubiquitous second messenger that regulates diverse physiological processes, including cell survival, proliferation, apoptosis, and autophagy, through interactions with downstream effector proteins (36, 37). Notably, Ca^2+^ signaling also plays critical roles in viral infections, particularly in IAV replication (38, 39). Previous studies have shown that Ca^2+^-calmodulin complexes contribute to transcriptional regulation during IAV infection (40), while inhibition of Ca^2+^ channels disrupts progeny virion assembly (41). While c-Fos expression is typically regulated by multiple signaling pathways and transcription factors, its regulation during IAV infection remained unclear. Building upon our finding that SERCA regulates c-Fos expression, and given SERCA’s role as the ER calcium pump that maintains cytosolic Ca^2+^ homeostasis and previous reports demonstrating that elevated cytosolic Ca^2+^ promote c-Fos transcription (34, 42), we hypothesized that IAV modulates cytosolic calcium levels to drive c-Fos expression. Our study provides compelling evidence that IAV upregulates c-Fos expression by increasing cytosolic Ca^2+^ levels. Specifically, we demonstrated that M2-mediated elevation of cytosolic Ca^2+^ is pivotal for c-Fos upregulation (Fig. 4), and the Ca^2+^ chelator BAPTA-AM suppresses c-Fos expression, autophagy, and viral replication (Fig. 4 and 5). These findings align with established Ca^2+^-dependent c-Fos induction mechanisms while extending this paradigm to viral infection. Importantly, we reveal that cytosolic Ca^2+^ promotes IAV replication through c-Fos upregulation, demonstrating how calcium signaling modulates viral replication.

Although the degradation pathways of IAV M2 protein have been partially characterized, current understanding remains incomplete. Previous studies established that M2 undergoes lysosomal degradation in HeLa cells mediated by the host factor MARCH8 (43). However, our investigation reveals a more complex scenario in which M2 is subject to dual regulation through both proteasomal and lysosomal pathways (Fig. 6G and H). This discrepancy likely reflects cell-type-specific regulation, as our experiments were conducted in 293H cells, distinct from the HeLa model used previously. Most significantly, we identify the host transcription factor c-Fos as a novel regulator of M2 stability. Through comprehensive analyses, we demonstrate that c-Fos interacts with M2 and protects it from both proteasomal and lysosomal degradation (Fig. 6I). Although c-Fos is canonically recognized for its transcriptional functions, our data uncover an important transcription-independent role in regulating M2 protein stability, which may contribute to viral replication. The mechanistic details of this protection will be an important focus of our future investigations. For instance, the E3 ubiquitin ligase MARCH8 is known to promote lysosomal degradation of M2 (43), and it is possible that c-Fos binding sterically hinders the recruitment of such factors.

Our findings align with a recent study by Gerodez et al. (18), which independently identified c-Fos as a proviral factor for IAV. Both studies consistently demonstrate that c-Fos knockdown impairs viral replication and reduces M2 protein expression. However, the mechanistic interpretations differ substantially. Gerodez et al. proposed that c-Fos acts primarily through nuclear transcriptional functions, including suppression of apoptosis and interferon-β, to support viral transcription. In contrast, our study uncovers a transcription-independent mechanism wherein c-Fos interacts directly with M2. By protecting M2 from both proteasomal and lysosomal degradation, c-Fos stabilizes this viral protein, thereby promoting autophagosome accumulation and facilitating viral replication. These divergent mechanisms are likely not mutually exclusive but may operate in parallel. Additionally, the distinct cell models employed across studies may also account for some of the observed discrepancies.

Current influenza antivirals primarily target viral proteins, including neuraminidase inhibitors (e.g., oseltamivir, zanamivir), polymerase inhibitors (e.g., baloxavir marboxil), and M2 ion channel blockers (e.g., amantadine, rimantadine) (44–47). However, the emergence of varying degrees of drug resistance to these agents (48–51) underscores the urgent need to identify novel antiviral targets through deeper investigation of IAV-host interactions. Our study demonstrates that IAV co-opts the host transcription factor c-Fos to stabilize viral M2 protein, thereby facilitating viral replication. This discovery reveals that disrupting the c-Fos-M2 interaction represents a promising antiviral strategy. Importantly, through molecular docking analysis, we predicted ten conserved M2 residues critical for the c-Fos-M2 interaction: ARG-18, ASN-20, SER-23, ASP-24, ASP-44, LYS-49, TYR-57, GLU-66, SER-71, and GLU-75 (Fig. S4A). To evaluate the translational potential of these findings, we analyzed the evolutionary conservation of these predicted interaction sites using 16,532 H1N1 (1930–2017), 2,813 H1N2 (1957–2017), 215 H2N2 (1957–2014), and 16,918 H3N2 (1967–2017) human IAV M2 protein sequences retrieved from the UniProt database. Among the ten identified residues, SER-23, ASP-24, ASP-44, LYS-49, GLU-66, SER-71, and GLU-75 exhibited pronounced evolutionary conservation across all four major human-infecting IAV subtypes (H1N1, H1N2, H2N2, and H3N2) (Fig. S4B). Given the functional importance of the c-Fos-M2 interaction in stabilizing M2 and promoting viral replication, and the high sequence conservation of these critical residues, the c-Fos-M2 interface represents an attractive target for developing broad-spectrum anti-influenza therapeutics with a high barrier to resistance.

In conclusion, our work establishes c-Fos as a critical host factor that promotes IAV replication through M2 stabilization and the consequent autophagosome accumulation. Small-molecule inhibitors disrupting c-Fos-M2 binding or modulators of calcium/c-Fos signaling could represent promising candidates for future antiviral development.

## MATERIALS AND METHODS

### Cell lines, viruses, and mouse strain

The human lung cancer cell lines NCI-H1395 (abbreviated as H1395) and NCI-H1975 (abbreviated as H1975) were cultured in RPMI 1640 medium (C11875500BT; Gibco) containing 10% fetal bovine serum (10270-106; Gibco) at 37°C and 5% CO_2_; 293H cells (used in previous studies and preserved in our laboratory) were cultured in Dulbecco’s modified Eagle medium (DMEM; C11995500BT; Gibco) containing 10% fetal bovine serum at 37°C and 5% CO_2_. The viral strains PR8 (A/Puerto Rico/8/1934) and WSN (A/WSN/1933) were preserved in our laboratory. The mouse strain used in this study was BALB/c.

### Antibodies, reagents, plasmids, and short interfering RNA (siRNA) oligonucleotides

The following primary antibodies were used: anti-LC3B (2775; Cell Signaling Technology, CST), anti-SQSTM1 (5114; CST), anti-GAPDH (2118; CST), anti-NP (10780-01; SouthernBiotech), anti-SERCA (A1097; ABclonal), anti-Flag (AE092; ABclonal), and anti-influenza A virus M2 (IT-003-015; Immune Technology). Horseradish peroxidase (HRP)-conjugated goat anti-rabbit (E030120-01; EarthOx) and anti-mouse (E030110-01; EarthOx) secondary antibodies were also employed. Key reagents included BAPTA-AM (HY-100545; MedChemExpress), T-5224 (B4664; APExBIO), cycloheximide (CHX, HY-12320; MedChemExpress), rapamycin (9904S; CST), MG132 (S2619; Selleck), bafilomycin A1 (A8510; Solarbio), and chloroquine (C6628; Sigma). siRNA oligonucleotides targeting ATP2A2 (5′-GGTGCTATTTACTACTTTA-3′), c-Fos (5′-TCTGCTTTGCAGACCGAGATT-3′; 5′-GTGGAACAGTTAUCTCCAGAA-3′ and 5′-CACTGCTTACACGTCTTCCTT-3′), and a negative control siNC (5′-TTCTCCGAACGTGTCACGT-3′) were synthesized by Synbio Technologies. Plasmids for mammalian expression of influenza A virus genes (PB2, PB1, PA, HA, NP, NA, M1, M2, NS1, NS2) were preserved in our laboratory.

### RNA-Seq analysis

Following total RNA extraction, mRNA was enriched using oligo(dT)-conjugated magnetic beads and subsequently fragmented at elevated temperature. The fragmented mRNA served as a template for first-strand cDNA synthesis via reverse transcription. During second-strand synthesis, the cDNA underwent end repair and adenine (A)-tailing. Sequencing adapters were then ligated to the fragments, which were purified and size-selected using selection beads. Finally, the library was amplified via PCR and sequenced on an Illumina NovaSeq 6000 platform.

### Real-time quantitative PCR (RT-qPCR) analysis

Total RNA from H1395 cells was extracted with the Universal RNA Purification Kit (EZB-RN4; EZBioscience) and reverse-transcribed into cDNA using HiScript III RT SuperMix. qPCR was performed using a 2× SYBR Green Master Mix (A0001; EZBioscience) on a LightCycler96 system (Roche) with the following protocol: 95°C for 30 s, then 40 cycles of 95°C for 10 s and 60°C for 30 s. Relative mRNA expression was normalized to GAPDH and calculated using the 2^–ΔΔCt method. The primers used in this study are listed in Table S1.

### Plaque assay

Viral titers were determined by plaque assay. Briefly, confluent MDCK monolayers in 12-well plates were inoculated with serially diluted viral supernatants (0.25 mL) and incubated at room temperature for 1 h with gentle swirling every 15 min. After the incubation, the cells were overlaid with 1 mL of culture medium containing 1% agarose and allowed to solidify at room temperature. The plates were then inverted and incubated at 37°C for 72 h. Finally, the cells were fixed and stained with 0.1% crystal violet after removal of the agarose overlay to visualize the plaques.

### Immunofluorescence assay and confocal fluorescence microscopy

Following treatments, cells were fixed, permeabilized, and blocked. They were then incubated with primary antibodies at 4°C overnight, followed by species-specific secondary antibodies (Alexa Fluor 488 or 594) and DAPI for nuclear staining. Images were acquired using an Olympus FV10i confocal microscope with sequential scanning. The number of LC3B puncta per cell was quantified blindly using ImageJ software.

### Flow cytometry

To measure cytosolic Ca^2+^ levels in H1395 and 293H cells, we used the fluorescent Ca^2+^ indicator Fluo-4 AM (F312, Dojindo). Cells were washed three times with PBS and loaded with 5 *μ*M Fluo-4 AM for 30 min. After incubation, the cells were harvested by centrifugation and analyzed via flow cytometry. For each sample, 30,000 events were recorded and used for subsequent analysis.

### Co-immunoprecipitation (Co-IP)

Cells in culture were washed twice with ice-cold PBS to completely remove residual medium. IP lysis buffer supplemented with protease inhibitor (Beyotime, ST507) was directly added onto cell layers and scraped on ice. Cell lysates were processed as mentioned above. For immunoprecipitation of FLAG-tagged proteins, cell lysates were incubated with anti-FLAG magnetic beads (MedChemExpress, HY-K0207) on a rotator at 4°C overnight. IP beads were then washed with IP lysis buffer four times, and boiled in 1× SDS loading buffer at 98°C for 5 min for SDS-PAGE and immunoblot analysis.

### Western blotting

Cells were lysed on ice and centrifuged to collect supernatants. Protein concentrations were determined using a BCA assay kit (23225; Thermo Scientific). After normalization, samples were boiled in SDS loading buffer, separated by SDS-PAGE, and transferred to PVDF membranes (IPVH00010; Millipore). The membranes were blocked with 5% non-fat milk and incubated with primary antibodies overnight at 4°C, followed by HRP-conjugated secondary antibodies at 37°C for 2 h. Protein bands were visualized using Clarity Western ECL substrate (170-5060; Bio-Rad).

### Cell viability assay

Cell viability was assessed using the Cell Counting Kit-8 (CCK-8; CK04, Dojindo) according to the manufacturer’s instructions. For drug treatment, H1395 cell monolayers were exposed to various concentrations of BAPTA-AM or T5224 for 24 h. For siRNA transfection, H1395 cells were transfected with siNC, siATP2A2, or sic-Fos, and cell viability was measured at 24 h post-transfection. In both assays, absorbance was read at 450 nm using a microplate spectrophotometer (BioTek), and cell viability was determined relative to negative control-treated cells.

### Protein degradation assay

293H cell lines were co-transfected with M2 plasmid with empty vector or Flag-c-Fos plasmid. At 24 h post-transfection, CHX (50 *μ*g/mL) was added to the medium and cells were harvested at different time points. Western blotting of cell lysates was performed to detect Flag-c-Fos, M2, and GAPDH. To investigate the cellular pathway of M2 degradation, MG132 (200 nM) or bafimycin A1 (2 nM) was added to the medium, and the cells were harvested 24 h after treatment. Three independent experiments were performed.

### Statistical analysis

Data in this study are expressed as the means ± standard deviations (SD), and differences were measured using Student’s *t* test. A P value of < 0.05 was considered statistically significant. For all analyses, the notations used to indicate significance between groups are the following: *, *P* < 0.05; **, *P* < 0.01; ***, *P* < 0.001; #, *P* > 0.05.

## Data Availability

The RNA-Seq data have been deposited in the GSA public database under the accession number HRA014120.

## SUPPLEMENTAL MATERIAL

Figure S1 jvi.00916-26-s0001.tif Cell viability assay after siRNA transfection.

Figure S2 jvi.00916-26-s0002.tif c-Fos promotes IAV replication in H1975 cells.

Figure S3 jvi.00916-26-s0003.tif Effect of c-Fos knockdown on seasonal influenza virus replication.

Figure S4 jvi.00916-26-s0004.tif Predicted interaction sites of c-Fos-M2 and alignment of M2 sequences.

Table S1 jvi.00916-26-s0005.docx Primers used for RT-qPCR.

ASM does not own the copyrights to Supplemental Material that may be linked to, or accessed through, an article. The authors have granted ASM a non-exclusive, world-wide license to publish the Supplemental Material files. Please contact the corresponding author directly for reuse.
