# Supplementary material for: c-Fos enhances influenza virus replication by stabilizing the M2 protein and promoting autophagosome accumulation
Source: J Virol. 2026 Jul 27;100(8):e00916-26. doi: 10.1128/jvi.00916-26 (PMC13483408; doi:10.1128/jvi.00916-26)
Supplement: Supplementary file 1 [file jvi.00916-26-s0005.docx]

**S1 Table.** The primers used for quantitative real-time PCR.

| **Name** | **Forward sequence (5’–3’)** | **Reverse sequence (5’–3’)** |
| --- | --- | --- |
| ATP2A2 | ATGGGGCTCCAACGAGTTAC | TTTCCTGCCATACACCCACAA |
| c-Fos | CCGGGGATAGCCTCTCTTACT | CCAGGTCCGTGCAGAAGTC |
| c-Fos (mouse) | CGGGTTTCAACGCCGACTA | TGGCACTAGAGACGGACAGAT |
| IAV-NP | TCAAGTGAGAGAGAGCCGGA | TCAAAGTCGTACCCACTGGC |
| IAV-M2 | AAACGAATGGGGGTGCAGAT | GTCAGCATCCACAGCACTCT |
| plasmid-M2 | ACACCTATCCGCAACGAGTG | CTTCCGGTACTCCTCCCTCA |
| GAPDH | GGAGCGAGATCCCTCCAAAAT | GGCTGTTGTCATACTTCTCATGG |
| GAPDH (mouse) | CGTCCCGTAGACAAAATGGT | TTGATGGCAACAATCTCCAC |
